# Induction of Apoptosis in Human Multiple Myeloma Cell Lines by Ebselen via Enhancing the Endogenous Reactive Oxygen Species Production

**DOI:** 10.1155/2014/696107

**Published:** 2014-01-27

**Authors:** Liang Zhang, Liwei Zhou, Jia Du, Mengxia Li, Chengyuan Qian, Yi Cheng, Yang Peng, Jiayin Xie, Dong Wang

**Affiliations:** Cancer Center, Research Institute of Surgery, Daping Hospital, Third Military Medical University, 10 Changjiang Zhi Road, Daping Yuzhong District, Chongqing 400042, China

## Abstract

Ebselen a selenoorganic compound showing glutathione peroxidase like activity is an anti-inflammatory and antioxidative agent. Its cytoprotective activity has been investigated in recent years. However, experimental evidence also shows that ebselen causes cell death in several cancer cell types whose mechanism has not yet been elucidated. In this study, we examined the effect of ebselen on multiple myeloma (MM) cell lines in vitro. The results showed that ebselen significantly enhanced the production of reactive oxygen species (ROS) accompanied by cell viability decrease and apoptosis rate increase. Further studies revealed that ebselen can induce Bax redistribution from the cytosol to mitochondria leading to mitochondrial membrane potential ΔΨm changes and cytochrome C release from the mitochondria to cytosol. Furtherly, we found that exogenous addition of N-acetyl cysteine (NAC) completely diminished the cell damage induced by ebselen. This result suggests that relatively high concentration of ebselen can induce MM cells apoptosis in culture by enhancing the production of endogenous ROS and triggering mitochondria mediated apoptotic pathway.

## 1. Introduction

Ebselen(2-phenyl-1,2-benzisoselenazol-3[2H]-one) is a selenoorganic compound exhibiting GSH and thioredoxin peroxidase like activity in vitro [1]. Due to its ability to scavenge reactive oxygen species (ROS), ebselen has been described as an anti-inflammatory and antioxidative agent which has potential chemoprevene effect on various diseases associated with oxidative stress. Previous studies had shown that ebselen can protect important organs or systems (cardiovascular [2], hepatic [3], gastrointestinal [4, 5], renal [2, 6], and neural systems [7–9]) from lipid peroxidation and oxidative damage. In many studies ebselen was shown to be an excellent substrate for human TrxR and Trx which established a novel antioxidant mechanism of ebselen as a direct substrate for Trx and TrxR and favoured this mechanism over the previously known glutathione peroxidase mimic in the presence of glutathione and glutathione reductase [10, 11].

In Contrast to inorganic selenium, the toxicity of ebselen is very low as the selenium atom in it is not bioavailable and cannot enter selenium metabolism in the organism [12]. However, a series of studies had reported that ebselen can also provoke cell death in several different human tumor cell types [13–16]; it shed some light on the new usage of ebselen as an antitumor agent. Although the mechanism underlying the toxicity induced by ebselen is not completely understood, previous studies had provided some proofs which indicated that apoptosis may play an important role in this process.

Apoptosis is one of the forms of cell death that plays a fundamental role in the development of multicellular organisms and numerous physiological processes. Imbalance between cell growth and apoptosis can lead to many pathological phenomena, including cancer [17, 18]. Intervening the apoptosis pathway is considered to be an effective strategy for cancer therapy. There is growing evidence that reactive oxygen species (ROS) who act as chemical messengers in cells play an important role in the process of apoptosis. Interestingly, ROS displays dual effect on apoptosis; that is to say, high level of ROS can induce apoptosis, while low concentration of ROS is essential for cell survival [19]. It has been widely accepted that many types of cancer cells bear more oxidative stress than normal cells, which may be critical for tumorigenesis, progress, and invasiveness [20, 21]. In view of the relationship of cancer, ROS, and apoptosis described above, we can formulate hypotheses that the antitumor activity of ebselen may due to the ROS production or elimination.

The main goals of this study is to shed light on the changing of ROS and other cellular events involved in apoptosis processes induced by ebselen. In our present work, we have examined the effect of ebselen on multiple myeloma (MM) cell lines; we found that ebselen can enhance the production of endogenous ROS, which further induce mitochondrial membrane potential lost and mitochondrial cytochrome C release by translocation of Bax protein into the mitochondria, ultimately triggered the mitochondrial-mediated apoptotic pathway.

## 2. Materials and Methods

### 2.1. Cell Culture and Reagents

The human multiple myeloma cell lines U266 and RPMI8226 were purchased from the American Type Culture Collection (Manassas, VA, USA). Both of the two cell lines were grown in RPMI 1640 medium supplemented with 10% fetal bovine serum, 100 U/mL penicillin, and 100 *μ*g/mL streptomycin (Hyclone, Logan, UT) and maintained at 37°C in a humidified atmosphere in the presence of 5% CO_2_-95% air.

Ebselen(2-phenyl-1,2-benzisoselenazol-3[2H]-one) was purchased from Sigma-Aldrich (St Louis, MO, USA), ROS Detection reagents Singlet ROS Probe was from Invitrogen (Carlsbad, CA, USA). The Cell Counting Kit-8, JC-1, and Cell Mitochondria Isolation Kit were from Beyotime.

### 2.2. Ebselen Treatment of MM Cells

Stock solutions of ebselen (10 mM) were prepared in DMSO. Cells were incubated in RPMI 1640 medium (with 5% FetalClone and antibiotics) containing the indicated amount of ebselen at 37°C in an atmosphere of 5% CO_2_. An amount of DMSO equivalent to that delivered with ebselen was added to the cells incubated in the absence of ebselen. N-Acetyl-L-cysteine (NAC; 15 mM in phosphate-buffered saline (PBS; 9.1 mM Na_2_HPO_4_, 1.7 Mm NaH_2_PO_4_, 150 mM NaCl, pH 7.4)), was added to the cells at the same time as ebselen.

### 2.3. Cell Counting Kit-8 Assay

Cells (5 × 10^3^) on 96-well plates were treated with ebselen as indicated. Cell counting kit-8 reagent was added to each well at a concentration of 1/10 volume, and the plates were incubated at 37°C for an additional 4 h. Absorbance was then measured at 450 nm as reference with a Microplate Reader 550 (Bio-Rad Laboratories, Hercules, CA). The following equation was used: cell viability (%) = OD value of treatment group/OD value of control group ×100%.

### 2.4. Cells Apoptosis Determination

MM cells were incubated in 60 mm plates and then treated with ebselen at various concentrations. After incubation at 37°C for the indicated time, cells were washed twice with PBS. Annexin V-propidium iodide (AV-PI) staining was then carried out using the AV-FITC Apoptosis Detection Kit (Biovision, Inc.). Briefly, the cells were resuspended in AV Binding Buffer at an appropriate density. AV and PI (2.5 mL each) were added; the samples were protected from light and incubated at room temperature for 15 min. Then analysis immediately using flow cytometry (FACScan, BD Bioscience, San Jose, CA, USA) was done.

### 2.5. ROS Production Measurement

The ROS production was measured following the manufacturer's instructions. Briefly, MM cells were incubated in 60 mm plates and then treated with ebselen at various concentrations. After incubation at 37°C for the indicated time, cells were washed twice with PBS and then incubated with 20 **μ**M Singlet ROS Probe in 0.2% BSA-PBS at 37°C for 25 min before immediate analysis using flow cytometry (FACScan, BD Bioscience, San Jose, CA, USA). The mean fluorescent intensity of 10000 analyzed cells in each treatment group was normalized by the mean fluorescent intensity of the control group of each cell line. The cells incubated with Singlet ROS Probe were also detected under laser scanning confocal microscope.

### 2.6. Mitochondrial Membrane Potential (MMP) Assay

The MMP was assessed using the JC-1 mitochondria staining kit for flow cytometry, following the manufacturer's recommendations. Briefly, cells were treated with various concentrations of photoirradiation for the indicated time then incubated in medium containing JC-1 probe (2.5–5 **μ**Gu/mL) for 30 min at 37°C. The cells were then incubated with 10 mM of JC-1 for 30 min at 378°C. After washing with ice-cold JC-1 binding buffer twice, MMP was measured immediately using flow cytometry. Two excitation wavelengths, 527 nm (green) and 590 nm (red), were used to detect the JC-1 monomer form and the JC-1 aggregate form, respectively. The red fluorescence was predominantly detected in healthy cells with high MMP, while its level was decreased in damaged mitochondria. The cells incubated with JC-1 were also detected under laser scanning confocal microscope.

### 2.7. Western Blot Assay and Antibodies

MM cells were treated with ebselen as indicated, then the cytosolic and mitochondrial extracts were obtained by the Cell Mitochondria Isolation Kit following the manufacturer's instructions. Proteins were then transferred to PVDF membranes (Bio-Rad, Hercules, CA). After blocking TBST (50 mM Tris-HCl, pH 7.5, 150 mM NaCl, and 0.1% (v/v) Tween 20) containing 5% (w/v) nonfat dry milk for 1 h at room temperature (RT), membranes were incubated with the specific primary antibody. After three washes with TBST, membranes were incubated for 1 h at RT with appropriate peroxidase conjugated secondary antibodies. Then, membranes were washed five times with TBST and the blots were reacted with chemiluminescent reagents and revealed with Biomax-Light films (Kodak, Rochester, NY). Suppliers and incubation conditions of antibodies used for Western blots were as follows: anti-BAX polyclonal (Gentext), overnight at 4°C, dilution 1 : 500; anti-Cytochrome C polyclonal (Gentext), dilution 1 : 500; anti-*β*-actin monoclonal (Sigma), 1 h at 37°C, dilution 1 : 2000.

### 2.8. Data Analysis

All results are presented as means ± SD of *n* observations, unless otherwise noted. Statistical significance was determined at the 95% confidence level using one-way ANOVA with Scheffe's test.

## 3. Result

### 3.1. Effect of Ebselen on MM Cell Viability

**Figure 1:**
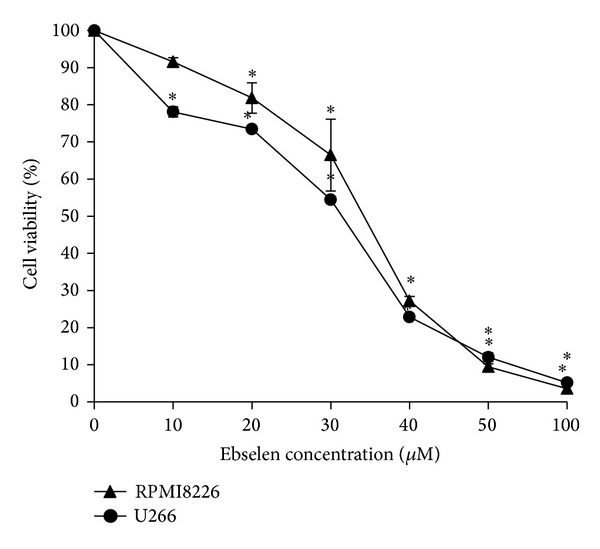
Concentration dependent changes of cell viability determined by CCK-8 assay in MM cells. Cells were treated with 0 (control), 10, 20, 30, 40, 50, and 100 *μ*M ebselen for 24 h, respectively. The significance was analyzed by one-way ANOVA. Data are presented as mean ± SD (*n* = 3). *Significant difference compared to the control group (*P*, 0.05, one-way ANOVA with Scheffe's test).

The concentration dependent changes of cell viability in ebselen treated MM cells, determined by CCK-8 Kit, are shown in Figure 1. The concentrations which we chose in this study are from 10 to 100 **μ**M; the lowest concentration used in this study (10 **μ**M) showed little effect on cell viability up to 4 h treatment. The ability of ebselen to decrease cell viability increased sharply when the concentration was up to 30 *μ*M. And the viability was reduced to less than 10% when the concentration reaches 100 *μ*M. On the basis of these results, we chose the concentration of 40 *μ*M (IC50) for further study.

### 3.2. Ebselen Can Induce MM Cell Apoptosis

**Figure 2:**
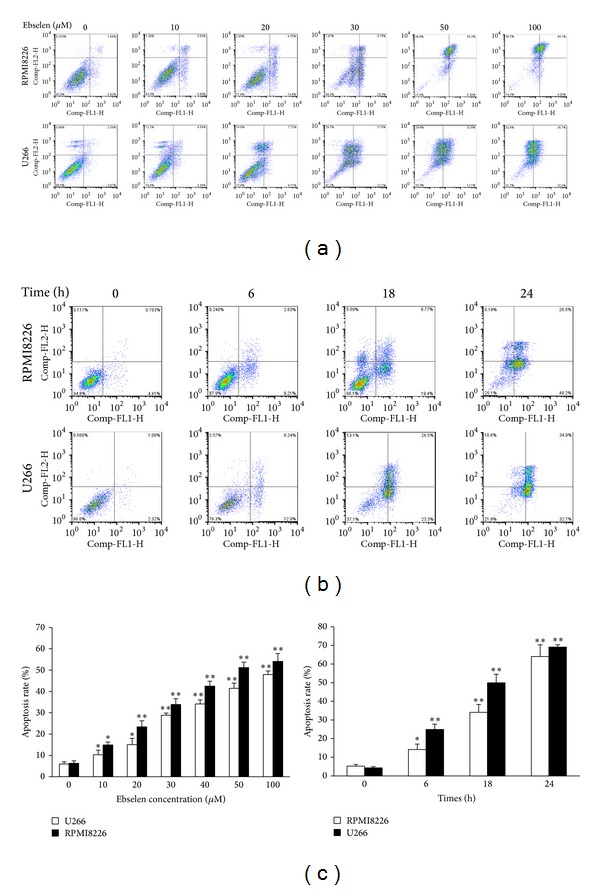
Ebselen induced apoptosis of MM cells analyzed by FACS, stained with annexin V-FITC/PI. (a) and (b) display the results of the cells treated with ebselen in a concentration and time dependent manner, respectively. The data is also showed in histogram (c). Data are presented as mean ± SD (*n* = 3). *Significant difference compared to the control group (*P < 0.05, **P < 0.01, one-way ANOVA with Scheffe's test).

To test the effects of concentration and time of ebselen on apoptosis of MM cells, we measured apoptosis by flow cytometry using Annexin V-FITC/PI staining. It was found that ebselen was able to increase the percentages of apoptosis of cells in a concentration (Figure 2(a)) and time (Figure 2(b)) dependent manner (Figure 2(c)). When MM cells were treated with 10 *μ*M ebselen for 24 hours, the apoptosis rate nearly doubled. The percentage of apoptotic cells was increased to 4 to 8 times when treated with 50 and 100 *μ*M ebselen. In the time-course study, cells were treated with 40 *μ*M ebselen for 6, 18, 24 hours, respectively, and then the data show that apoptosis rate was significantly increased with time.

### 3.3. The Production of ROS Was Increased in Ebselen Treated MM Cells

**Figure 3:**
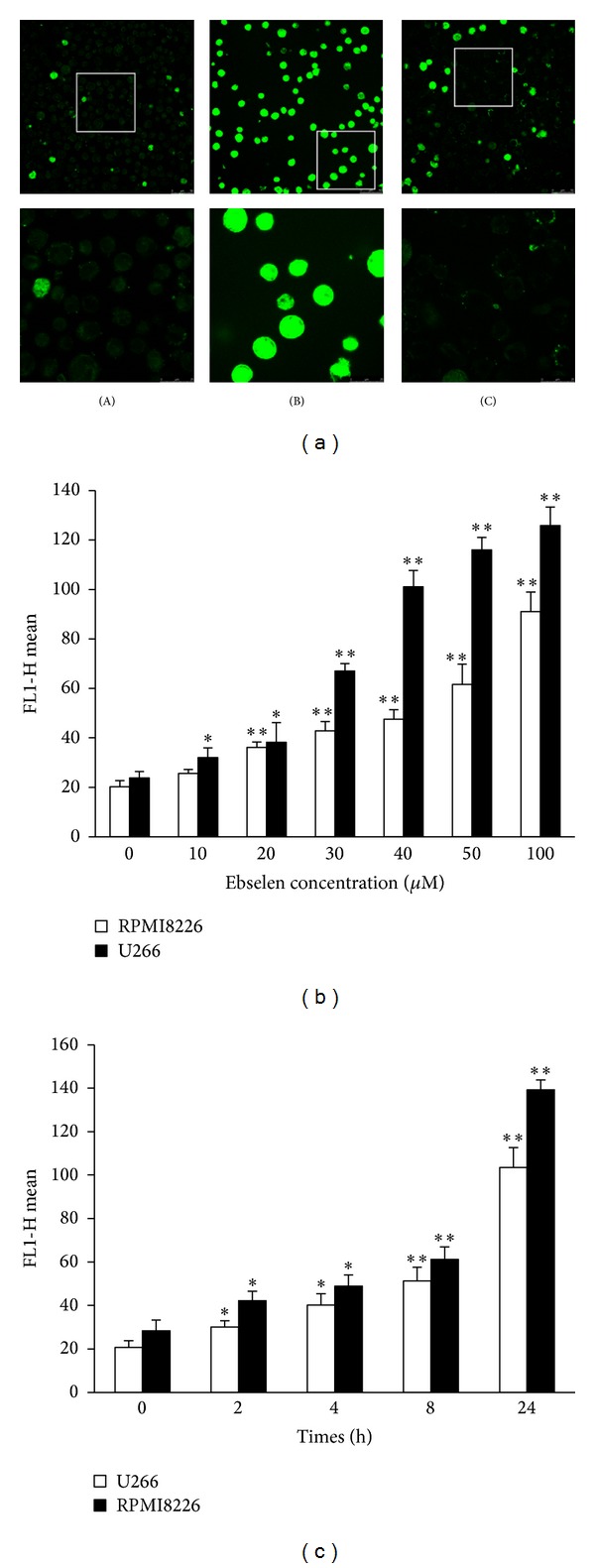
The ROS production of MM cells treated with ebselen in presence or absence of NAC was determined by Singlet ROS Probe. Fluorescence images of Singlet ROS Probe loaded cells were obtained under laser confocal microscopy ((A) DMSO, (B) ebselen 40 *μ*M, and (C) ebselen 40 *μ*M + NAC 15 mM). The average of fluorescence intensity obtained under FACS shows that ebselen induced ROS increasing of MM cells in a concentration (b) and time (c) manner. Each value represents the mean ± SD from three independent experiments. *Significant difference compared to the control group (*P < 0.05, **P < 0.01, one-way ANOVA with Scheffe's test).

The preceding data indicated that ebselen is showed to be significantly toxic to MM cells. To determine whether the ebselen induced cytotoxic effects were mediated through oxidative stress, we measured the production of ROS in cell cultures challenged with ebselen in time and concentration manners. Then we found that, 4 h after treatment with different concentration ebselen, U266 and RPMI8226 cells demonstrated an increased level of ROS production (Figure 3(b)). After being treated with 40 *μ*M ebselen for 2, 4, 8, and 24 hours, respectively, the production of ROS in cells increased markedly (Figure 3(c)).

### 3.4. Ebselen Can Redistribute the Location of Bax in MM Cells

**Figure 4:**
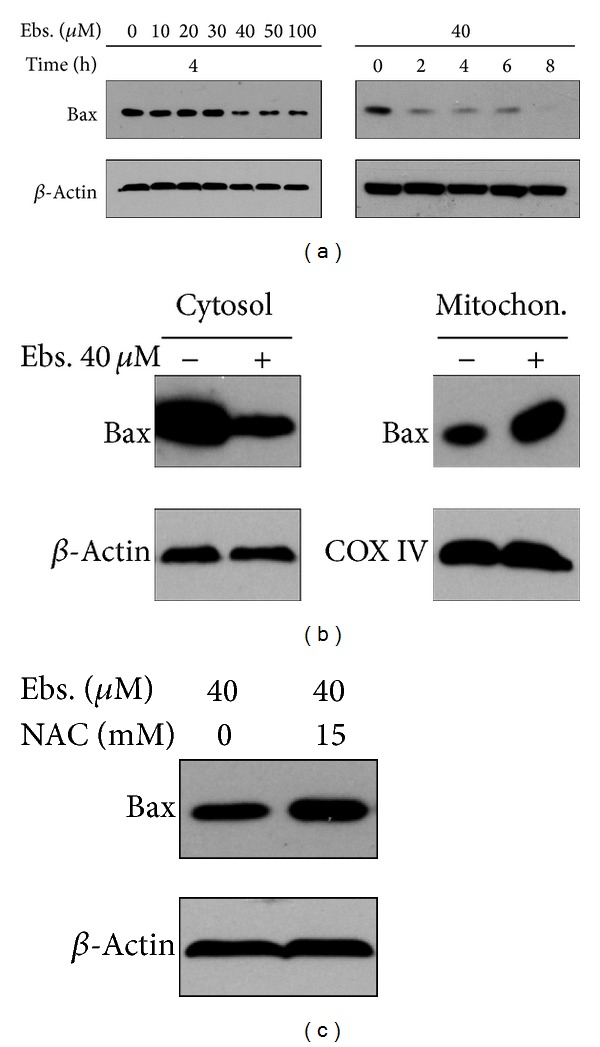
Bax translocation to mitochondria from cytoplasm after being treated with ebselen is determined by Western blot. (a) Ebselen decrased Bax level in cytoplasm in a concentration and time dependent manner. (b) Mitochondria Bax level increased and cytoplasmic Bax level decreased in MM cells treated with ebselen 40 *μ*M for 4 hours. (c) The decreasing of cytoplasmic Bax level can be recovered partially by 15 mM NAC.

Bax belongs to Bcl-2 family which is involved in apoptosis; there are some works which reported that Bax can be translocated to mitochondria after a cytotoxic stimulus [22]. So the next set of experiments were designed to find out if the Bax protein in MM cells behaved as others described after being treated with ebselen. Then we measured Bax by Western blot, including its expression and location. The result indicated that the Bax levels in the cytosol fraction were decreased after ebselen treatment in a concentration and time dependent manner (Figure 4(a)). After 40 *μ*M ebselen treatment of MM cells for 4 hours, the Bax levels increased significantly in mitochondrial fraction and, in contrast, decreased in cytosol fraction (Figure 4(b)), which mean that ebselen can induce Bax translocation to mitochondria from cytosol.

### 3.5. Effect of Ebselen on Mitochondrial Membrane Potential

**Figure 5:**
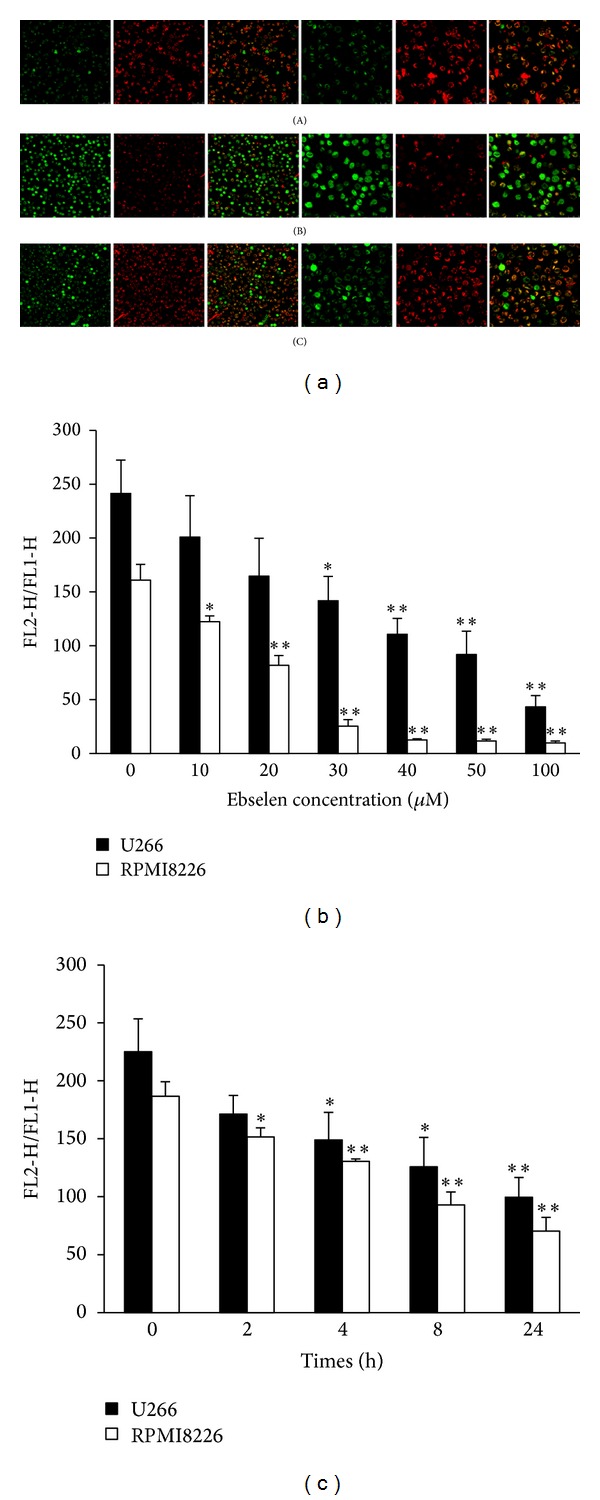
Mitochondrial membrane potential of MM cells analyzed by laser confocal microscopy and FACS after being loaded with JC-1. (a) Fluorescence images of JC-1 Probe loaded cells were obtained under laser confocal microscopy ((A) DMSO, (B) Ebselen 40 *μ*M, and (C) ebselen 40 *μ*M + NAC 15 mM). The ratio of red (FL2-H) and green (FL1-H) mean fluorescence intensity obtained under FACS shows ebselen induced MMP lost of MM cells in a concentration (b) and time (c) manner. Each value represents the mean ± SD from three independent experiments. *Significant difference compared to the control group (*P < 0.05, **P < 0.01, one-way ANOVA with Scheffe's test).

Mitochondrial membrane potential (ΔΨm) is a marker of mitochondrial function which is closely related with mitochondrial membrane permeability. To determine whether MM cells apoptosis induced by ebselen was associated with mitochondrial dysfunction, we measured the MMP of MM cells treated with ebselen as indicated by flow cytometry using JC-1 probe in this study. The results show the mean fluorescence intensity ratio of red fluorescence and green fluorescence (FL2-H/FL1-H) which indicated that the MMP levels decreased significantly after ebselen treatment in a concentration and time dependent manner (Figures 5(b) and 5(c)). Thus, we got some evidence to assume that ebselen induced MM cell apoptosis via mitochondrial pathway.

### 3.6. Ebselen Induced Cytochrome C Release from the Mitochondria to Cytosol

**Figure 6:**
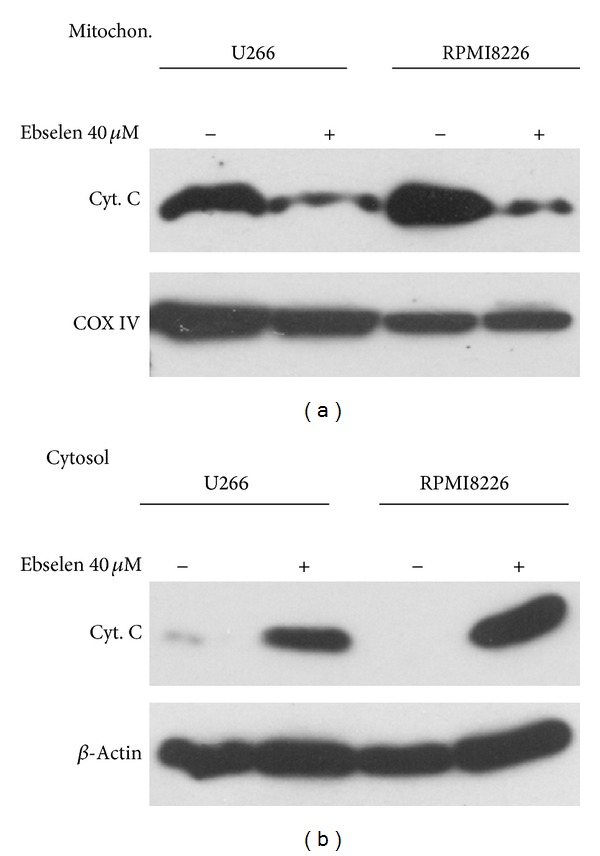
Ebselen induced mitochondrial cytochrome C release from the mitochondria to cytosol. A representative immunoblot of MM cell cultures in presence or absence of ebselen showing levels of cytochrome C in cytosolic and mitochondrial extracts. Cells were treated for 4 h with ebselen. COX-IV and *β*-actin protein levels were used as internal reference of mitochondrial and cytosol, respectively. The record shown is representative of three experiments.

Mitochondrial cytochrome C release to the cytosol is considered to be one of the key steps in cell death pathways. In order to ascertain if this occurred in our study, levels of cytochrome C were determined by Western blot in cytosolic fractions and mitochondrial fractions, respectively, from cell culture treated with 40 **μ**M ebselen for 4 hours. The result shows that cell cultures challenged with 40 *μ*M ebselen for 4 hours were accompanied with cytochrome C release from the mitochondria to cytosol (Figure 6).

### 3.7. N-Acetyl Cysteine (NAC) Protected MM Cells from Apoptosis Induced by Ebselen

**Figure 7:**
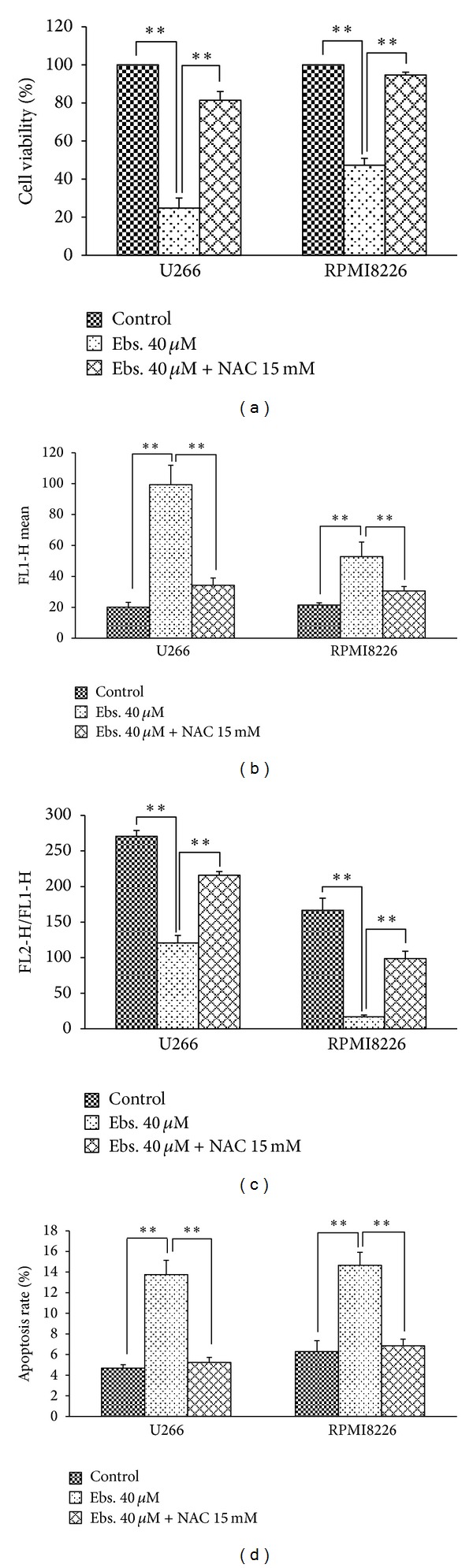
The damage of MM cells induced by ebselen was attenuated by NAC. (a) The cell viability decreased by 40 *μ*M ebselen treatment for 24 hours was recovered by 15 mM NAC. (b) The increasing of ROS in MM cells treated with 40 *μ*M for 4 hours was significantly reduced by 15 mM NAC. (c) 15 mM NAC resumed the MMP loss induced by 40 *μ*M ebselen. (d) The apoptosis rate increased by 40 *μ*M ebselen was attenuated by 15 mM NAC. Each value represents the mean ± SD from three independent experiments. *Significant difference compared to the control group (*P < 0.05, **P < 0.01, one-way ANOVA with Scheffe's test).

To investigate the role of ROS in ebselen-induced cell apoptosis, we pretreated MM cells with an efficient ROS scavenger, NAC, followed by concomitant 40 *μ*M ebselen exposure for 4 hours. The presence of 15 mM NAC markedly protected MM cells against ebselen-induced series of pathological events above-mentioned. The cell viability decreasing (Figure 7(a)), enhancement of ROS production (Figures 3(a) and 7(b)), MMP losing (Figures 5(a) and 7(c)), apoptosis rate increasing (Figure 7(d)), and Bax translocation (Figure 4(c)), all of these events induced by ebselen, can be restored by NAC.

## 4. Discussion

Multiple myeloma (MM) is a deadly plasma cell cancer that resides in the bone marrow. The key clinical features of MM include the production of a monoclonal paraprotein, amyloidosis, renal insufficiency, anemia, increased BM angiogenesis, osteolytic bone lesions, severe bone pain, spontaneous fractures, and hypercalcemia [23]. Although high-dose chemotherapy and autologous stem cell transplantation improved survival in younger patients, the natural history of MM has been changed with the availability of five new agents approved in last 10 years (thalidomide, bortezomib, lenalidomide, liposomal doxorubicin, and carfilzomib). Despite this significant improvement in overall outcome, MM remains incurable in the majority of patients prompting continued search for additional therapeutic options.

Ebselen is part of the National Institutes of Health Clinical Collection, a chemical library of bioavailable drugs considered clinically safe but without proven use [24]. Though Meotti and his colleagues' study added to our understanding of the potential renal and hepatic toxicity of ebselen [25], more literatures indicated that ebselen was clinically safe for human beings [26, 27]. On the other hands, it is well established that ebselen has potent cytotoxic activity against many different human cancer cell lines, whose precise mechanism often varies depending on the cancer cell type. Yang et al. observed that ebselen induced apoptosis of HepG2 cells through a mechanism that involves intracellular thiol depletion and mitochondrial permeability transition [15]. Guérin and Gauthier reported that ebselen induced the rapid necrotic cell death of Sp2/0-Ag14 hybridoma cells [16]. Sharma et al. work indicated that ebselen sensitizes glioblastoma cells to tumor necrosis factor (TNF-*α*) induced apoptosis through two distinct pathways involving NF-*κ*B downregulation and Fas mediated formation of death inducing signaling complex [28]. Recently, Maraldiy et al. revealed that ebselen has the capability of induction of apoptosis in a human acute myeloid leukemia cell line M07e via inhibition of ROS generation [29].

In current study, ebselen exhibited cytotoxicity to multiple myeloma cell lines, U266 and RPMI8226, in a concentration and time dependent manner. The production of ROS in cells increased sharply followed by Bax translocation and MMP depolarization under ebselen challenge. Meanwhile, cytochrome C was released from the mitochondria and then triggered the cell apoptosis. Previous studies have established ebselen as a potent inhibitor of the mitochondrial complexes. The complexes I and II could be considered important molecular targets of ebselen after exposure to high dosages [30–33]. In consistence with previous studies, we found that ebselen treatment induced mitochondria dysfunction and cytochrome C release which is one of the key steps in apoptosis pathways. Our study extends these findings by elucidating the manner in which ebselen activated this particular event. Our data revealed that cytochrome C release occurred indirectly, via the redistribution of Bax, rather than via mitochondrial PTP formation or direct mitochondrial damage. Because Morin et al. had shown that, at low concentrations, ebselen acts as an inducer of PTP opening, at higher concentrations, it acts as an inhibitor in isolated mitochondria [34]. Bax is a proapoptotic protein that plays an important role in apoptotic pathways. When inactivated, Bax can be found as monomers in the cytosol or loosely associated with the outer mitochondrial membrane. Once activated, Bax inserts into the mitochondrial outer membrane [35], forms pores by oligomerization with Bak which further cause the release of the contents of the mitochondrial intermembrane space, including cytochrome C, into the cytosol [36].

The question then arises as to the nature of the mechanism involved in ebselen-induced Bax activation. We consider ROS as more relevant second messengers in Bax activation. We found that ebselen induced an increase in ROS formation besides Bax activation. Further study showed that these effects were significantly blocked when the cells were pretreated with N-acetyl cysteine (NAC), a specific ROS inhibitor. So we can deduce that ROS are upstream events in ebselen induce apoptotic process of MM cells. As a traditional antioxidative agent, the mechanism of ebselen increasing intracellular ROS generation is still not clear. There are numerous reports in the literature indicating that the biological activities of ebselen are dependent on the intermediates and products from the biotransformation process. The biotransformation process of ebselen which contains many steps and different outcomes is very complicated. Thiol plays an important role in this process as its capacity can influence the outcomes. In the presence of excess thiol, ebselen showed free radical scavenger activity by consuming reduced thiol. When the concentration of the thiol is relatively low, ebselen consumes thiol without decomposing peroxide [37–39]. The thiol is critical for cells to maintain redox balance [40]; thus the relatively high concentration of ebselen exhibits prooxidant activity by consuming thiol without decomposing peroxide. In view of that mitochondria is a major intracellular source of reactive oxygen species (ROS), which are mainly generated at complexes I and III of the respiratory chain; ebselen may increase ROS production by inducing mitochondrial dysfunction. Puntel and his colleagues had investigated the effect of ebselen on mitochondrial complexes activity and got the conclusion that ebselen can induce mitochondrial dysfunction by oxidizing critical thiol groups from mitochondrial complexes I and II [41].

Over the last decade, many reports revealed that phytochemicals targeting ROS metabolism can selectively kill cancer cells by raising the level of ROS above a toxic threshold. Since cancer cells show higher levels of endogenous ROS compared with their normal cells, the toxic threshold can be easily achieved in cancer cells [42, 43]. A series of studies had revealed that ebselen can attenuate the side effect of many classical antitumor agents such as cisplatin [44–50], daunorubicin [51], cyclophosphamide [52], and doxorubicin [53]. More encouragingly, Lynch et al. and Baldew et al. showed us that, combined with cisplatin or cis-diamminedichloroplatinum, ebselen cannot only reduce the side effects but also enhance the antitumor activity of these drugs [50, 54]. Thus, ebselen seemed to be promising candidate for antitumor therapy.

## 5. Conclusion

In this study, our data revealed that ebselen induced MM cell apoptosis, mediated by increased ROS production. These deleterious conditions resulted in the translocation of Bax and the release of cytochrome C. All of the new observations may provide further elucidation of the mechanism of pharmacological (anticancer) and toxicological effects of ebselen.
